# Prevalence and clinical/molecular characteristics of *PTEN* mutations in Turkish children with autism spectrum disorders and macrocephaly

**DOI:** 10.1002/mgg3.1739

**Published:** 2021-07-16

**Authors:** Hande Kaymakcalan, İlyas Kaya, Nagihan Cevher Binici, Emrah Nikerel, Burcu Özbaran, Mehmet Görkem Aksoy, Seda Erbilgin, Gonca Özyurt, Noor Jahan, Didem Çelik, Kanay Yararbaş, Leyla Yalçınkaya, Sezen Köse, Sibel Durak, Adife Gulhan Ercan‐Sencicek

**Affiliations:** ^1^ Pediatric Genetics Unit Department of Pediatrics Demiroglu Bilim University Istanbul Turkey; ^2^ Department of Child and Adolescent Psychiatry Istanbul University Istanbul Faculty of Medicine Istanbul Turkey; ^3^ Department of Child and Adolescent Psychiatry Dr Behcet Uz Child Disease and Surgery Training and Research Hospital Istanbul Turkey; ^4^ Department of Bioinformatics Yeditepe University Istanbul Turkey; ^5^ Department of Child and Adolescent Psychiatry Ege University Faculty of Medicine Izmir Turkey; ^6^ Department of Child and Adolescent Psychiatry Prof. Dr. Cemil Tascioglu City Hospital Istanbul Turkey; ^7^ Department of Child and Adolescent Psychiatry Izmir Katip Celebi University Faculty of Medicine Izmir Turkey; ^8^ Department of Medical Genetics Demiroglu Bilim University Istanbul Turkey; ^9^ Department of Molecular Biology and Genetics Bilkent University Faculty of Science Ankara Turkey; ^10^ Masonic Medical Research Institute Utica New York USA; ^11^ Yale University School of Medicine New Haven Connecticut USA; ^12^ Department of Neurosurgery Program on Neurogenetics New Haven Connecticut USA

**Keywords:** autism spectrum disorder, macrocephaly, mutation, prevalence, *PTEN*

## Abstract

**Background:**

Phosphatase and tensin homolog (*PTEN*) germline mutations are associated with cancer syndromes (*PTEN* hamartoma tumor syndrome; PHTS) and in pediatric patients with autism spectrum disorder (ASD) and macrocephaly. The exact prevalence of *PTEN* mutations in patients with ASD and macrocephaly is uncertain; with prevalence rates ranging from 1% to 17%. Most studies are retrospective and contain more adult than pediatric patients, there is a need for more prospective pediatric studies.

**Methods:**

We recruited 131 patients (108 males, 23 females) with ASD and macrocephaly between the ages of 3 and 18 from five child and adolescent psychiatry clinics in Turkey from July 2018 to December 2019. We defined macrocephaly as occipito‐frontal HC size at or greater than 2 standard deviations (SD) above the mean for age and sex on standard growth charts. *PTEN* gene sequence analysis was performed using a MiSeq next generation sequencing (NGS) platform, (Illumina).

**Conclusion:**

*PTEN* gene sequence analyses identified three pathogenic/likely pathogenic mutations [NM_000314.6; p.(Pro204Leu), (p.Arg233*) and novel (p.Tyr176Cys*8)] and two variants of uncertain significance (VUS) [NM_000314.6; p.(Ala79Thr) and c.*10del]. We also report that patient with (p.Tyr176Cys*8) mutation has Grade 1 hepatosteatosis, a phenotype not previously described. This is the first *PTEN* prevalence study of patients with ASD and macrocephaly in Turkey and South Eastern Europe region with a largest homogenous cohort. The prevalence of *PTEN* mutations was found 3.8% (VUS included) or 2.29% (VUS omitted). We recommend testing for *PTEN* mutations in all patients with ASD and macrocephaly.

## Graphical abstract

This is the first PTEN prevalence study of patients with ASD and macrocephaly in Turkey and South Eastern Europe region with a largest homogenous cohort. The prevalence of PTEN mutations was found 3.8% (VUS included) or 2.29% (VUS omitted). We report for the first time a patient with PTEN mutation with hepatosteatosis.

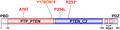

## 1 INTRODUCTION

Phosphatase and tensin homolog (*PTEN*) (OMIM 601728) is a tumor suppressor negatively regulates Phosphoinositide 3‐kinase/AKT/mammalian target of rapamycin (PI3K/AKT/mTOR) pathway that plays an important role in cell growth, survival, and proliferation (Lv et al., 2013). Germline pathogenic variants in the *PTEN* gene lead to a range of clinical outcomes including cancer syndrome phenotypes collectively known as PTEN hamartoma tumor syndrome (PHTS), and autism spectrum disorder (ASD) with macrocephaly (MIM 605309) (Lv et al., 2013). Indeed, in mice model of PTEN haploinsufficiency, overgrowth of brain is detectable from birth to adulthood (Chen et al., 2015).

It had been reported that the frequency of *PTEN* mutations ranged from 1% to 22% in patients with ASD and macrocephaly (Buxbaum et al., 2007; Conti et al., 2012; Frazier et al., 2015; Herman et al., 2007; Hobert et al., 2014; Klein et al., 2013; Kurata et al., 2018; McBride et al., 2010; Varga et al., 2009). Even though same *PTEN* mutation in different individuals lead to different phenotype (Leslie & Longy, 2016), missense mutations were predominantly reported in autism and macrocephaly syndrome (Leslie & Longy, 2016; Spinelli et al., 2015). These mutations lower, but do not abolish, *PTEN’s* key activity (Smith et al., 2019). Mighell et al., (2018) proposed that mutations associated with ASD and developmental delay are unstable, but more catalytically active than mutations causing PHTS. It had been suggested that *PTEN* mutation carrier ASD patients have a distinct neurobehavioral phenotype compared to idiopathic ASD (Busch et al., 2019) that strongly suggests the importance of reliable genotype‐phenotype studies to help in patient management, prognosis and therapeutic selection by identifying key mutations associated to ASD phenotypes. Since clinical testing guidelines for PTHS in children are fairly new and not applied uniformly (Butler et al., 2005; Hansen‐Kiss et al., 2017; Macken et al., 2019), PTHS often goes undetected in children. We hope that our study will increase awareness of this rare disease in Turkey.

In this study, we screened *PTEN* variants in children with ASD, mild intellectual disability and macrocephaly without significant developmental delay in Turkey to determine the prevalence of *PTEN* mutations in pediatric ASD and macrocephaly patients and to find novel mutations that would lead to greater insight into genotype‐phenotype correlations for *PTEN* mutations.

## 2 METHODS

After Istanbul University ethics committee approval (Number: 2014/798), we recruited 131 Turkish children (108 males, 23 females) aged 3–18 years with macrocephaly and ASD who were seen at five different child and adolescent psychiatry clinics in Turkey from July 2018 to December 2019. ASD diagnosis was made by experienced child and adolescent psychiatrists using DSM V criteria. We defined macrocephaly as occipito‐frontal HC size at or greater than 2 standard deviations (SD) above the mean for age and sex on standard growth charts. All patients had thyroid ultrasounds (USG) and cranial magnetic resonance imagining (MRI).

Total of 3cc's of peripheral venous blood was collected from each patient after written informed parental consent forms were signed. The blood samples were archived and stored for possible further analysis. DNA extraction was performed with DNA extraction kits (Qiagen. inc), and the DNA samples were preserved at −20°C for future analysis.

First tier *PTEN* gene sequence analysis was performed using a MiSeq next generation sequencing (NGS) platform, (Illumina, San Diego, CA, USA) an FDA approved diagnostic system. All coding exons of the *PTEN* gene and their flanking splice site junctions were amplified by in house designed primers. PCRs were validated by using agarose gel electrophoresis. After PCR amplification, the libraries were prepared with the NexteraXT kit (Illumina Inc.), according to the manufacturer's instructions. Next‐generation sequencing was carried on MiSeq (Illumina Inc.). Sequences were aligned to the hg19 genome within MiSeq Reporter software (Illumina Inc.). Visualization of the data was performed with IGV 2.3 (Broad Institute) software. Confirmatory Sanger sequencing was performed for 10 randomly selected patients, patients with positive results, and their respective parents.

To determine the confidence interval of the estimated prevalence, we followed Lwanga and Lemeshow (1991), where the absolute precision is defined as: *D* = sqrt(z^2^ × *P* × (1 − *P*)/*n*) with *z* the significance threshold, *P* the calculated prevalence, *D* the absolute precision and n the sample size. Using the genomic data on 131 patients and considering five SNPs discovered, we calculated the prevalence to be 3.8 ± 3.3%, with 95% confidence interval being (0.5, 7.1). The clinical significance of each variant was evaluated with ClinVar (http://www.ncbi.nlm.nih.gov/clinvar/) database and Database of Genomic Structural Variation (dbVar).

Patients with positive results were seen by a pediatric geneticist (except for patient 3 lost to follow up) for dysmorphology exam, additional family history, and genetic counselling.

## 3 RESULTS

In our cohort of 131 patients (108 males, 23 females) between the ages of 3 and 18, we found two variants of uncertain significance and three pathogenic mutations according to ClinVar database. The prevalence of *PTEN* mutations is 3.8% and 2.29% by including or excluding VUS, respectively.

Among pathogenic mutations, two are frameshift mutations and one is a missense mutation. We confirmed the *de novo* nature of these mutations by using DNA from the parents of four patients. Clinical and molecular characteristics of these patients are summarized in Table 1.

**TABLE 1:** Clinical and molecular characteristics of *PTEN* positive patients

Pt	Mutation	ClinVar	dbSNP	Age	Sex	Psychiatric comorbidities	Type of mutation	Thyroid usg	Cranial MR	Cutaneous lesions	HC at exam/SD	Dysmorphology
1	c.611C>T p.(P204L)	NA	—	5 years	F	—	Likely pathogenic	normal	Nonspecific hyperintense areas in T2	—	53,5 cm/+2.01	Macrocephaly
2	c. 235G>A p.(Ala79Thr)	Likely benign	Uncertain significance	8 years	M	ADHD	VUS	normal	normal	—	56cm/+2.42	Almond shaped eyes, low set ears,prominent ear lobes, macrocephaly
3^x^	C* 10del	Uncertain significance	—	7 years	M	—	VUS	NA	NA	NA	55cm/+2.41	Macrocephaly
4	c.697C>T p.(Arg233*)	Pathogenic with no conflicts	Pathogenic with no conflicts	5 years	F	ID	Pathogenic	normal	Nonspecific hyperintense areas in T2	—	60cm/+5.65	Macrocephaly
5	c.525_526dupTG p.(Tyr176Cys*8)	—	—	15 years	M	—	Likely pathogenic	normal	normal	Penile freckling	61cm/+ 2.81	Frontal bossing, macrocephaly

Note

3^x^: Patient lost contact.

Abbreviations: HC, head circumference; ID, Intellectual disability; NA, not available VUS, variant of unsignificance; Patient, Pt; SD, standard deviation; usg, ultrasound.

Patient 1 was a 5‐year‐old female diagnosed with mild ASD when she was 4 years old. She did not have any dysmorphic features nor other medical problems. She had normal intellectual capacity based on observations and Turkish developmental test (Savasir et al., 2005). She did not have cutaneous lesions. Her weight and height were −0.4 and −0.5 SD respectively whereas her HC was +2.01 SD. She had thyroid and abdominal USG’s which were normal. Cranial MRI showed nonspecific hyperintense areas on T2.). Her *PTEN* mutation has moderate evidence level according to Clingen PTEN Expert Panel phenotype scoring. (Mester et al.,l., 2018) (Table 2 and SuppInfo 1).

**TABLE 2:** PTEN variant classification according to Clingen PTEN Expert Panel

Pt	Phenotype specificity score	Phenotypic evidence level	Variant classification
1	2	Moderate	PS1, PS2, PM5, PS4_M, PP2, PP3
2	4	Strong	PS4, PM1, PP3
3^x^	4	Strong	PS4, BP7
4	5	Strong	PVS1, PS2, PS4
5	7	Strong	PVS1, PS2, PS4

Note

3^x^: Patient lost contact.

Abbreviations: Please refer to SuppInfo 1.

She carries a *de novo* P204L [Ref seq NM_000314.6; c.611C>T p.(Pro204Leu)] missense variant that was predicted to be pathogenic according to multiple in silico algorithms (Table 1 and SuppInfo 2).

Patient 2 was an 8‐year‐old male diagnosed with mild ASD when he was 5 years old. He also had attention deficit hyperactivity disorder (ADHD). He had mild intellectual deficiency based on observations and Turkish developmental test. On physical exam, he was found to have almond shaped eyes, low set ears, and prominent ear lobes. His HC was 56cm, 99. 32% and +2.41 SD. He did not want his height and weight measured during exam. Thyroid USG and cranial MRI was normal. There were no cutaneous lesions. His *PTEN* mutation has strong evidence level according to Clingen PTEN Expert Panel phenotype scoring. (Table 2 and SuppInfo 1) A79T [Ref seq NM_000314.6; c.235G>A, p.(Ala79Thr)] missense variant was identified in this patient. The dbSNP database classified this variant as of uncertain significance, while ClinVar classified it as likely benign (Table 1 and SuppInfo 2).

Patient 3 was a 7‐year‐old male diagnosed with mild ASD when he was 3 years old. His HC was 55cm, 98.07% and +2.42 SD. He and his family were lost to follow up. Developmental test could not be done. He had mild ID based on observations. His *PTEN* mutation has strong evidence level according to Clingen PTEN Expert Panel phenotype scoring. (Table 2 and SuppInfo 1) c.*10del (Ref seq NM_000314.6; c.*10del) variant was identified. It was classified as a variant of uncertain significance in the ClinVar database (Table 1 and SuppInfo 2).

Patient 4 was a 5‐year‐old female diagnosed with mild ASD when she was 3 years old. She had mild intellectual deficiency based on observations and Turkish developmental test. Her HC had the biggest SD among 131 patients; measuring 59 cm, 99.98% and +5.65 SD. Her weight was +3.47 SD. She had a normal EEG and thyroid USG. Cranial MRI showed nonspecific hyperintense areas on T2. She was non‐ dysmorphic. There were no cutaneous lesions. Her *PTEN* mutation has strong evidence level according to Clingen PTEN Expert Panel phenotype scoring. (Table 2 and SuppInfo 1) Her mother also had HC +2 SD, a nasal bridge lipoma, diabetes mellitus, and hypertension. She was suffering from depression. A *de novo* p.Arg233* [Ref seq NM_000314.6; c.697C>T, (p.Arg233*)] loss‐of‐function (LOF) mutation was identified. It was classified as pathogenic with no conflicts in ClinVar and dbSNP database (Table 1 and SuppInfo 2).

Patient 5 was a 15‐year‐old male diagnosed with mild ASD when he was 7 years old. He had mild intellectual deficiency according to observations. His parents refused the developmental test. His HC was 61 cm, 99.75% and +2.81 SD. His weight was −1.05 SD and height was +0.38 SD. He had frontal bossing and penile freckling. Thyroid and scrotal USG were normal. Abdominal USG showed grade I hepatosteatosis. Cranial MRI was normal. His *PTEN* mutation has strong evidence level according to Clingen PTEN Expert Panel phenotype scoring. (Table 2 and SuppInfo 1) This patient fit the diagnostic criteria of BRRS. A novel *de novo* p.Y176Cfs*8 [Ref seq NM_000314.6; c.525_526dup, (p.Tyr176Cys*8)] LOF mutation was found. It was classified as likely pathogenic in ACMG classification. (Table 1 and SuppInfo 2) It has not previously been reported in the literature nor found in the Population Frequency Databases.

## 4 DISCUSSION

**FIGURE 1:**
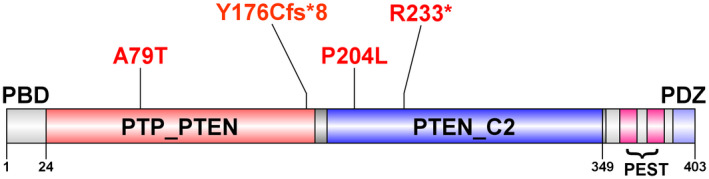
Mutations within the functional domain structure of human PTEN (with modification from Yehia and Eng (2018)) PBD: a phosphatidylinositol‐4,5‐bisphosphate (PIP2)‐binding domain; PTEN_C2: C2 domain of PTEN tumor‐suppressor protein; PTP_PTEN: Dual specificity phosphatase, catalytic domain; PEST: (proline, glutamic acid, serine, threonine)

*PTEN* is composed of 403 amino acids that comprise five functional domains: a phosphatidylinositol4,5‐bisphosphate (PIP2)‐binding domain (PBD) and a phosphatase domain containing the catalytic core (spans amino acids 123–130) at the N‐terminus, a C2 domain, two PEST (proline, glutamic acid, serine, threonine) domains, and a PDZ interaction motif for protein‐protein interactions at the C‐terminus (Yehia & Eng, 2018) (Figure 1).

The c.*10del (NM_000314.6; c.*10del) variant (rs756681683) that is located in 3’UTR region of the *PTEN* gene is classified as a variant of uncertain significance in the ClinVar database. It was absent from controls in Exome Sequencing Project, 1000 Genomes Project, or Exome Aggregation Consortium. However, in ALFA Project (Allele Frequency Aggregator), the minor allele frequency reported for the variant was below 1% (delT=0.00036) (Phan et al., 2020) (Table 1 and SuppInfo 2).

Novel loss‐of‐function mutation p.Y176Cfs*8 (NM_000314.6; c.525_526dup, p.Tyr176Cys*8) and p.Arg233* [NM_000314.6; c.697C>T, (p.Arg233*)], located in PTP and C2 domains of *PTEN*, respectively, result in an early truncated protein. They might affect the enzymatic activity and protein stability of *PTEN*, cell migration, and protein–protein interactions (Phan et al., 2020; Song et al., 2011; Vazquez et al., 2000). p.Y176Cfs*8 meets criteria to be classified as likely pathogenic (Table 1 and SuppInfo 2). The other LOF mutation of *PTEN* c.697C>T at cDNA level creates a stop codon from Arginine at 233 position at the protein level. It was classified as pathogenic with no conflicts in ClinVar database (ClinVar: 7813) and it is a well‐known stop codon mutation reported in the literature multiple times and results in Cowden Syndrome, Bannayan‐Riley‐Ruvalcaba syndrome and other cancers (Busch et al., 2013; Lachlan et al., 2007; Marsh et al., 1997; Ngeow et al., 2014).

**FIGURE 2:**
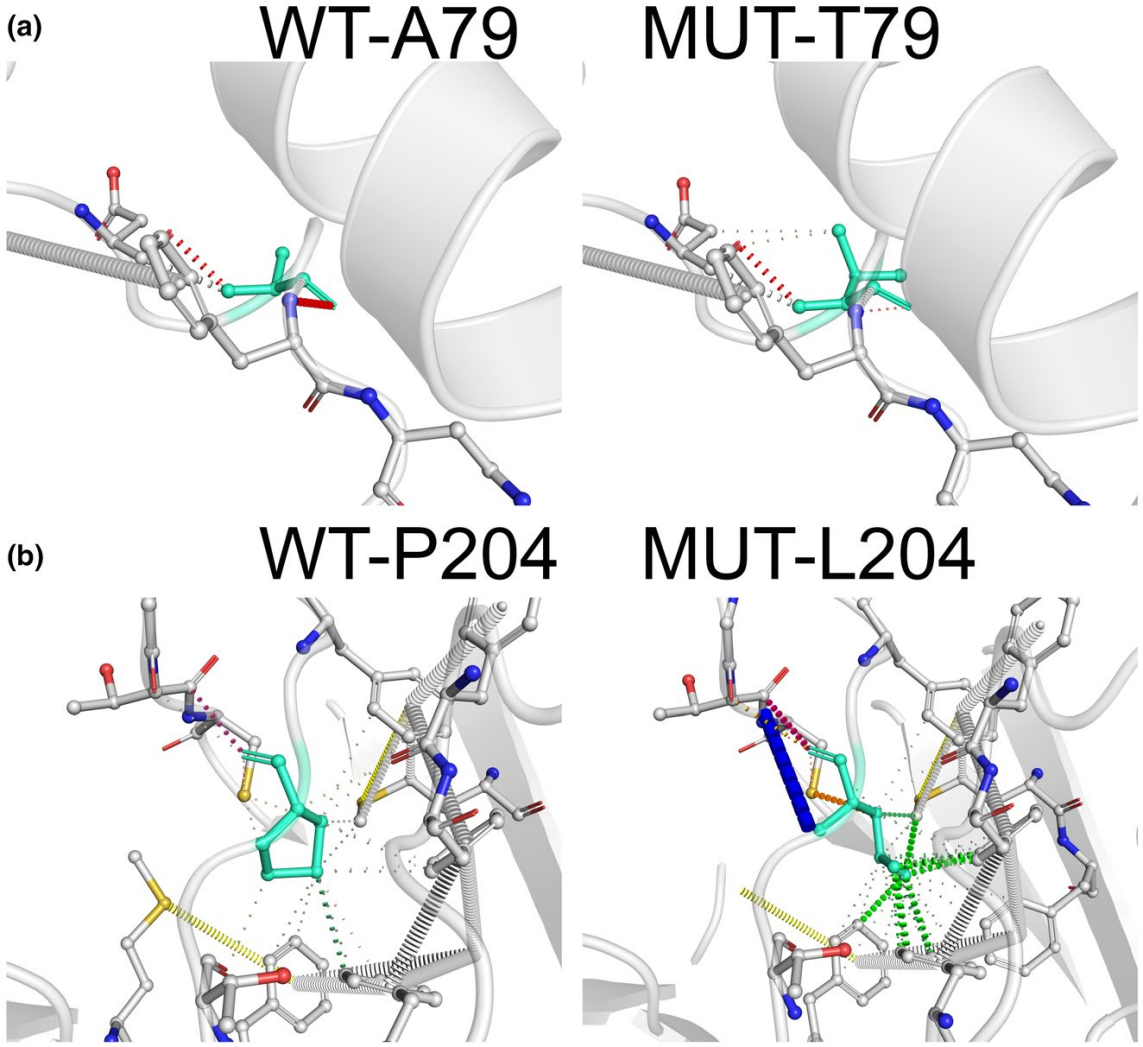
(a) Structural alteration of the wild‐type residue A79 by the mutant T79 illustrated by DynaMut. (b) Structural alteration of the wild‐type residue P204 by the mutant L204 illustrated by DynaMut. Wild‐type and mutant residues are colored in light‐green

The A79T]NM_000314.6; c.235G>A, p.(Ala79Thr)] variant was reported with low frequency (0.01%) in gnomAD exomes and ExAC. The dbSNP database classified this variant as of uncertain significance, while ClinVar classified it as likely benign (dbSNP: rs202004587, ClinVar: 41682). It was predicted to be deleterious by multiple in silico algorithms (MetalR, MetaSVM and FATHMM), while some of the predictions obtained from SIFT, REVEL, Polyphen suggested a benign effect on protein function (Table 1 and SuppInfo 2). *PTEN* is a gene that has a low rate of benign missense variation and where missense variants are a common mechanism of disease (Raftopoulou et al., 2004). The A79T variant was located on the catalytic phosphatase tensin‐type domain (spans amino acids 14 – 185). The analysis of the effect of the A79T substitution on the stability and conformational dynamics of the protein (PDB:1D5R) using DynaMut web server suggested that A79 destabilizes this protein (ΔΔG: −0.3431 kcal/mol; a negative value of ΔΔG indicates the mutation destabilizes the protein) (Wu et al., 2000) (Figure 2a). Additionally, we used HOPE web server (Project Have yOur Protein Explained) that analyzes the structural and functional effects of point mutations (Rodrigues et al., 2018). Wildtype A79 residue positioned within PTP domain is smaller and more hydrophobic than the mutant T79 residue that suggests mutation of theA79T might disturb the function of the protein.

P204L [NM_000314.6; c.611C>T p.(Pro204Leu)] missense variant was identified in our study, it was previously reported in cancer database. In addition, this variant was predicted to be deleterious by PolyPhen, FATHMM, ClinPred, MetaSVM, REVEL, and SIFT in silico analyses. An alternative P204A (p.Pro204Ala) variant has been found to be Likely Pathogenic in the ClinVar Database (ClinVar: 189415) (Table 1 and SuppInfo 2). Also, additional missense variants in nearby residues (F200S, T202I, M205V, S207R) have been reported in *PTEN*‐related disorders (Stenson et al., 2014), providing functional importance of this region of the protein. Based on the currently available evidence, P204L is, therefore, considered likely pathogenic. The analysis of the effect of P204L missense mutation that is located at the C2 domain using DynaMut web server found the positive ΔΔG (ΔΔG: 1.022 kcal/mol), which indicates the mutation does not destabilize the protein. However, HOPE web server revealed that the mutant residues of P204L bigger than the wild‐type residue (proline) and the mutation introduces an amino acid (leucine) with different properties, which can disturb the C2 domain and abolish its function. Moreover, rigidity of a protein structure is essential for specific function. The wild‐type 204 residue, proline, is known to be very rigid (Figure 2b) and substitution with leucine can disrupt this required rigidity of the *PTEN* protein.

Macrocephaly prevalence in ASD is estimated to be 20% in some studies (Fombonne et al., 1999; Miles et al., 2000), however another study reported no difference in head size between children with autism and controls (Langen et al., 2009). Although it was not the aim of our study, we found a 10.2% prevalence of macrocephaly in one center. At this center, head circumferences of 361 patients with autism spectrum disorder were measured for our study and 37 of these patients had macrocephaly. (SuppInfo 3) To the best of our knowledge this is the largest cohort of pediatric patients with ASD and macrocephaly and is the first prevalence study of *PTEN* mutations in macrocephaly and ASD in Turkey and South Eastern Europe region.

The patient 5 with *de novo* pathogenic LOF mutation (c.525_526dup, p.Tyr176Cys*8) displayed Grade 1 hepatosteatosis. *PTEN* loss has been previously hypothesized to cause hepatosteatosis by resulting in increased lipogenesis and hepatic apoB‐lipoprotein degredation (Qiu et al., 2008). Clinically, our patient does not have any other risk factors for hepatosteatosis (patient is not obese, does not have diabetes, does not have high cholesterol and not an adult) Therefore, *PTEN* mutation in this patient may be also associated with hepatosteatosis. This feature, to the best of our knowledge, is not previously described in the literature. Clinical outcome of pediatric patients with *PTEN* mutations are not well known yet; there are few studies suggesting a follow up plan in this group (Ciaccio et al., 2019). Smpokou et al., (2015) showed that thyroid carcinoma can occur at a very early age (6–7 years). This indicates the importance of screening for *PTEN* mutations to allow later thyroid carcinoma surveillance. Ciaccio et al., (2019) recommend screening of patients with ASD when HC is more than 3 SD. However, in our cohort, three patients with *PTEN* mutations have HC less than 3 SD. Also a progressive increase in HC is described. (Balci et al., 2018; Vanderver et al., 2014) We therefore recommend screening all patients with ASD whose HC are more than 2 SD.

Studies showed that individuals with ASD who carries *PTEN* mutations have reduced performance on attention, impulsivity, reaction time, processing speed, motor coordination and worse ID’s compared to individuals with ASD without *PTEN* mutations (Busch et al., 2013, 2019; Frazier et al., 2015). The limitations of our study is that we could not do a detailed neurobehavioral tests to observe these domains. Interestingly, in our cohort, one patient did not have ID and others had mild ID. However, it is not possible to make a genotype‐phenotype correlation at this time.

Studies of the mutation types in *PTEN* have varying results (Spinelli et al.,; 2015) and the genotype‐phenotype correlations reported are not substantial enough to predict the phenotype. (Macken et al.,; 2019).

Among our pathogenic mutations, two are LOF mutations and one is missense mutation. With this small number it is difficult to make a conclusion. Patient 5 with LOF mutation, demonstrated the clinical phenotype of BRRS. Patient 4 with LOF mutation had the biggest head circumference and since she is only 5 years old she may not show the clinical characteristics of PHTS yet. (She carries a well‐known stop codon mutation reported in the literature multiple times and results in Cowden Syndrome, Bannayan‐Riley‐Ruvalcaba syndrome and other cancers).

Most of the PHTS‐linked PTEN mutations are loss‐of‐function mutations (Rademacher & Eickholt, 2019) and our findings are in line with this hypothesis although as stated by Macken et al in their paper, in the absence of observational studies we cannot predict the phenotypes of children into adulthood. Due to this lack of firm genotype‐phenotype correlations, children with pathogenic or likely pathogenic *PTEN* variants are advised to follow PHTS cancer surveillance guidelines.

Identification of *PTEN* mutations is important for accurate genetic counselling, patient follow up, management and treatment with targeted therapies on the horizon.

## CONFLICT OF INTEREST

Authors declare no conflict of interest.

## AUTHOR CONTRIBUTIONS

H.K designed and supervised the study and wrote the manuscript. E.N performed data and statistical analysis. K.Y performed genetic analysis. I.K, N.C, B.Ö, S.E, G.Ö, S.G, and S.D made ASD diagnosis. M.G.A, N.J, and D.Ç took the consents, did the HC measurements and collected peripheral blood sample. L.Y did the literature review. A.G.E.S assisted in analyzing the genetic data and reviewed the manuscript.

## Supporting information

Supplementary Material

Supplementary Material

Supplementary Material
