# Supplementary material for: Prevalence and clinical/molecular characteristics of PTEN mutations in Turkish children with autism spectrum disorders and macrocephaly
Source: Mol Genet Genomic Med. 2021 Jul 16;9(8):e1739. doi: 10.1002/mgg3.1739 (PMC8404225; doi:10.1002/mgg3.1739)
Supplement: Supplementary file 1 — Supplementary Material [file MGG3-9-e1739-s002.docx]

**Reference:**

<https://www.ncbi.nlm.nih.gov/pmc/articles/PMC6329583/>

**Summary:**

Patient 1:

2 points, Moderate Evidence Level (based on phenotypic scoring)

PS1, PS2, PM5, PS4_M, PP2, PP3

Patient 2:

4 points, Strong Evidence Level

PS4, PM1, PP3, BP4

Patient 3:

4 points, Strong Evidence Level

PS4, BP7

Patient 4:

5 points, Strong Evidence Level

PVS1, PS2, PS4

Patient 5:

7 points, Strong Evidence Level

PVS1, PS2, PS4

**PTEN Phenotype Scoring**

Patient 1

Macrocephaly of >2 SD to <4 SD : 2 points

Total: 2 points

MODERATE EVIDENCE LEVEL

Patient 2

Macrocephaly of >2 SD to <4 SD : 2 points

Autism/developmental delay (DD)/intellectual disability (ID) : 2 points

Total: 4 points

STRONG EVIDENCE

Patient 3

Macrocephaly of >2 SD to <4 SD : 2 points

Autism/developmental delay (DD)/intellectual disability (ID) : 2 points

Total: 4 points

STRONG EVIDENCE

Patient 4

Extreme macrocephaly (≥4 SD) : 3 points

Autism/developmental delay (DD)/intellectual disability (ID) : 2 points

Total: 5 points

STRONG EVIDENCE

Patient 5

Macrocephaly of >2 SD to <4 SD : 2 points

Autism/developmental delay (DD)/intellectual disability (ID) : 2 points

Penile freckling: 3 points

Total : 7 points

Strong Evidence

**Criteria**

Patient 1

*PS1*

Same amino acid change as a previously established pathogenic variant regardless of nucleotide change OR different variant at same nucleotide position as a pathogenic splicing variant, where in silico models predict impact equal to or greater than the known pathogenic variant.

*PS2*

De novo (both maternity and paternity confirmed) in a patient with the disease and no family history.

PM5
Missense change at an amino acid residue where a different missense change determined to be pathogenic or likely pathogenic has been seen before. In addition, variant being interrogated must have a BLOSUM62 score equal to or less than the known variant.

PS4_M

Proband specificity score of 2-3.5 (see text).

PP2

Missense variant in a gene that has a low rate of benign missense variation and in which missense variants are a common mechanism of disease.

PP3
Multiple lines of computational evidence support a deleterious effect on the gene or gene product. To be applied only to synonymous or intronic variants where at least 2 out of 3 *in silico* models predict a splicing impact.

Patient 2

PS4

Use 2: Proband specificity score of 4-15.5 (see text).

PM1

Located in a mutational hot spot and/or critical and well-established functional domain. Defined to include residues in catalytic motifs: 90-94, 123-130, 166-168 ([NP_000305.3](https://www.ncbi.nlm.nih.gov/protein/NP_000305.3)).

PP3
Multiple lines of computational evidence support a deleterious effect on the gene or gene product. To be applied only to synonymous or intronic variants where at least 2 out of 3 *in silico* models predict a splicing impact.

BP4

Multiple lines of computational evidence suggest no impact on gene or gene product (conservation, evolutionary, splicing impact, etc.). To be applied only to synonymous or intronic variants where at least 2 out of 3 in silico models predict no splicing impact.

BP6

Reputable source recently reports variant as benign, but the evidence is not available to perform an independent evaluation.

Patient 3

PS4

Use 2: Proband specificity score of 4-15.5 (see text).

BP7

A synonymous (silent) or intronic variant at or beyond +7/−21 (5’/3’ exonic) for which splicing prediction algorithms predict no impact to the splice consensus sequence nor the creation of a new splice site AND the nucleotide is not conserved.

Patient 4

PVS1

Null variant (nonsense, frameshift, canonical ± 1 or 2 splice sites, initiation codon, single or multi-exon deletion) predicted to result in nonsense-mediated decay or causing truncation/frameshift at or 5’ to c.1121 ([NM_000314.4](https://www.ncbi.nlm.nih.gov/nuccore/NM_000314.4)).

PS2

De novo (both maternity and paternity confirmed) in a patient with the disease and no family history.

PS4

Use 2: Proband specificity score of 4-15.5 (see text).

Patient 5

PVS1

Null variant (nonsense, frameshift, canonical ± 1 or 2 splice sites, initiation codon, single or multi-exon deletion) predicted to result in nonsense-mediated decay or causing truncation/frameshift at or 5’ to c.1121 ([NM_000314.4](https://www.ncbi.nlm.nih.gov/nuccore/NM_000314.4)).

PS2

De novo (both maternity and paternity confirmed) in a patient with the disease and no family history.

PS4

Use 2: Proband specificity score of 4-15.5 (see text).
